# MdVQ17 negatively regulates apple resistance to Glomerella leaf spot by promoting MdWRKY17-mediated salicylic acid degradation and pectin lyase activity

**DOI:** 10.1093/hr/uhae159

**Published:** 2024-06-07

**Authors:** Dingyue Duan, Jie Yang, Ran Yi, Qinglong Dong, Mingrui Shi, Jingxuan He, Ke Mao, Fengwang Ma

**Affiliations:** State Key Laboratory for Crop Stress Resistance and High-Efficiency Production/Shaanxi Key Laboratory of Apple, College of Horticulture, Northwest A & F University, Yangling 712100, Shaanxi, China; State Key Laboratory for Crop Stress Resistance and High-Efficiency Production/Shaanxi Key Laboratory of Apple, College of Horticulture, Northwest A & F University, Yangling 712100, Shaanxi, China; State Key Laboratory for Crop Stress Resistance and High-Efficiency Production/Shaanxi Key Laboratory of Apple, College of Horticulture, Northwest A & F University, Yangling 712100, Shaanxi, China; State Key Laboratory for Crop Stress Resistance and High-Efficiency Production/Shaanxi Key Laboratory of Apple, College of Horticulture, Northwest A & F University, Yangling 712100, Shaanxi, China; State Key Laboratory for Crop Stress Resistance and High-Efficiency Production/Shaanxi Key Laboratory of Apple, College of Horticulture, Northwest A & F University, Yangling 712100, Shaanxi, China; State Key Laboratory for Crop Stress Resistance and High-Efficiency Production/Shaanxi Key Laboratory of Apple, College of Horticulture, Northwest A & F University, Yangling 712100, Shaanxi, China; State Key Laboratory for Crop Stress Resistance and High-Efficiency Production/Shaanxi Key Laboratory of Apple, College of Horticulture, Northwest A & F University, Yangling 712100, Shaanxi, China; State Key Laboratory for Crop Stress Resistance and High-Efficiency Production/Shaanxi Key Laboratory of Apple, College of Horticulture, Northwest A & F University, Yangling 712100, Shaanxi, China

## Abstract

Glomerella leaf spot (GLS) is a fungal disease caused by *Colletotrichum fructicola*, which severely restricts the yield and quality of apples. Valine–glutamine (VQ) proteins are transcriptional regulators involved in the regulation of plant growth and stress responses. However, little is known about the role of VQ proteins in the biotic stress response in apple. Here, a VQ gene, *MdVQ17*, that was highly induced by *C. fructicola* infection was identified. Overexpression of *MdVQ17* in apple increased susceptibility to *C. fructicola* and significantly reduced the salicylic acid content and β-1,3-glucanase and chitinase activities. Based on yeast two-hybrid screening, MdWRKY17, which promotes susceptibility to *C. fructicola*, was identified as an MdVQ17-interacting protein. Co-expression of MdVQ17 can promote the binding and transcriptional activation activity of MdWRKY17 on the promoter of *Downy Mildew Resistant 6* (*MdDMR6*), thereby promoting MdWRKY17-mediated salicylic acid degradation. Based on DNA affinity purification sequencing, the pectin lyase-encoding gene *MdPL-like* was identified as a direct target of MdWRKY17. MdWRKY17 can directly bind to the promoter of *MdPL-like* and activate its transcription, and the binding and activation of MdWRKY17 on the *MdPL-like* promoter were significantly enhanced by MdVQ17 co-expression. Functional identification showed that MdPL-like promoted pectin lyase activity and susceptibility to *C. fructicola*. In sum, these results demonstrate that the MdVQ17-MdWRKY17 module mediates the response to *C. fructicola* infection by regulating salicylic acid accumulation and pectin lyase activity. Our findings provide novel insights into the mechanisms by which the VQ–WRKY complex modulates plant pathogen defense responses.

## Introduction

Apple (*Malus × domestica* Borkh.) is widely grown in temperate regions worldwide; it is highly popular among consumers for its unique flavor and rich nutrient profile. In recent years, Glomerella leaf spot (GLS) has become an increasingly significant problem in multiple apple-producing areas in China, and this poses a major threat to the sustainable and healthy development of China’s apple industry [1]. GLS is mainly caused by the dominant species of the hemibiotrophic nutritional pathogen *Colletotrichum fructicola* [1]. GLS infects leaves and fruits under high temperature and humidity conditions, which causes leaf death and fruit spots and induces fruit fall, and this has a major effect on apple fruit yield and quality [2, 3]. GLS also weakens apple tree vigor, which decreases fruit yield the following year. The most effective method for controlling GLS is the application of dithiocarbamate fungicides [3]. However, the long-term use of such fungicides leads to ecological deterioration, poses fruit safety and quality issues, and significantly increases production costs [2, 3]. Therefore, in order to develop new methods of prevention and control, it is crucial to elucidate the molecular mechanisms underlying apple response to GLS infection. Such studies can also provide important optimized genes and technical support for improving and cultivating new apple disease-resistant germplasm through molecular breeding methods.

Plant-specific valine–glutamine (VQ) family proteins are transcriptional regulatory cofactors that contain a conserved VQ (FxxhVQxhTG) motif [4]. The conserved VQ motif plays an important role in transcriptional activity, subcellular localization, and protein interaction [4–6]. There is increasing evidence that VQ proteins regulate plant tolerance to pathogens. For example, *vq12 vq29* mutant plants exhibit markedly enhanced resistance to *Botrytis cinerea*, while transgenic *Arabidopsis* plants overexpressing *AtVQ12* or *AtVQ29* are sensitive to *B. cinerea* [7]. The *sigma factor-binging protein 1* (*sib1*)/*vq23* mutation reduces resistance to *Pseudomonas syringae*, and constitutive overexpression of *SIB1/VQ23* can enhance resistance to *P. syringae* [8]. A study of *VQ21/Mitogen-activated protein kinase4 substrate1* (*MKS1*) found that the resistance of plants overexpressing *AtVQ21/MKS1* to *P. syringae* was significantly enhanced [9], but the resistance to *B. cinerea* was decreased [10, 11]. In rice, *OsVQ13*-overexpressing transgenic plants showed increased resistance to *Xanthomonas oryzae* pv. *oryzae* (*Xoo*) [12]. Overexpression of *GmVQ58* inhibited resistance of *Arabidopsis* mutant to the common cutworm (CCW), but silencing *GmVQ58* in soybean roots increased the resistance to CCW [13]. In tomato, *SlVQ15-*overexpressing transgenic plants showed enhanced resistance to *B. cinerea*, while silencing *SlVQ15* increased susceptibility to *B. cinerea* [14]. At present, studies of VQ proteins in apple mainly focus on abiotic stress responses [15–18]. It is unclear what role they play in regulating defense responses.

Various studies have indicated that VQ proteins can interact with various functional proteins or transcription factors (TFs) to fine-tune plant growth and stress responses [14, 19–23]. For example, AtVQ29 can interact with phytochrome-interacting factor1 (PIF1), which regulates the expression of *xyloglucan endotransglycosylase7* (*XTR7*), which is a gene associated with cell elongation, and affects the early seedling morphogenesis of *Arabidopsis* [19]. *Arabidopsis* and rice VQ proteins mediate the response to pathogens by interacting with mitogen-activated protein kinase (MAPKs) [4, 20, 22]. In *Arabidopsis*, VQ22/jasmonate-associated VQ motif gene 1 (JAV1) was found to interact with WRKY51 and WRKY28 to negatively regulate jasmonic acid (JA)-mediated plant defense [23]. In addition, SlVQ15 physically interacts with SlWRKY31 to synergistically regulate tomato resistance to *B. cinerea* [14]. WRKY TFs play important roles in plant growth and stress responses [24, 25]. WRKY–VQ interactions have been extensively studied in *Arabidopsis*, soybean, banana, and apple plants [26–28]. However, the VQ–WRKY complex in woody plants has been examined in just a few studies for its biological functions and molecular mechanisms.

Pectin is the main component of the cell wall. Pectate lyase or pectin lyase promotes the cracking and elimination of de-esterified pectin [29]. Many studies indicate that *pectate lyase-like* genes (*PLL*s) have multiple biological functions, and they are mainly involved in flower organ development, fruit ripening and softening, and defense responses. *Arabidopsis powdery mildew-resistance 6* (*pmr6*) mutant plants show enhanced resistance to powdery mildew due to vegetative growth inhibition caused by changing the composition of the cell wall [30]. *AtPLL18* and *AtPLL19* play important roles in the development and maintenance of syncytia [31]. Knockdown of *OsPLL3* can disrupt the development of mature pollen during the pollen dehiscence stage, which leads to pollen abortion and affects the development of floral organs and spikelets in rice [32]. The tomato *SlPL* gene is significantly upregulated during fruit softening. Inhibiting the expression of *SlPL* reduces the pectate lyase activity in fruit, increases the cellulose and hemicellulose content, and alters the size, number, and thickness of pericarp cells, which leads to improved fruit hardness [33].

In apple, MdWRKY17 activates the expression of the salicylic acid (SA) degradation-related gene *Downy Mildew Resistant 6* (*MdDMR6*) to reduce the endogenous SA content, which results in reduced resistance to *C. fructicola* [3]. In this study, we investigated the expression patterns of different apple *MdVQ* genes following infection with GLS. Among the *MdVQ* genes analyzed, we observed a notable upregulation of *MdVQ17* in response to GLS infection. Functional identification of *MdVQ17* in transgenic apple leaves indicates that it negatively regulates GLS tolerance. As an MdWRKY17-interacting protein, MdVQ17 promotes the expression of *MdDMR6* and the pectin lyase-encoding gene *MdPL-like* by promoting the promoter binding and transcriptional activation activity of MdWRKY17, which reduces the SA content, increases pectin lyase activity, and increases susceptibility to *C. fructicola*. These results provide novel insights into the mechanism underlying the response to GLS infection in apple via the MdVQ17–MdWRKY17–*MdDMR6/MdPL-like* module.

## Results

### Gene cloning and characterization of *MdVQ17*

**Figure 1:**
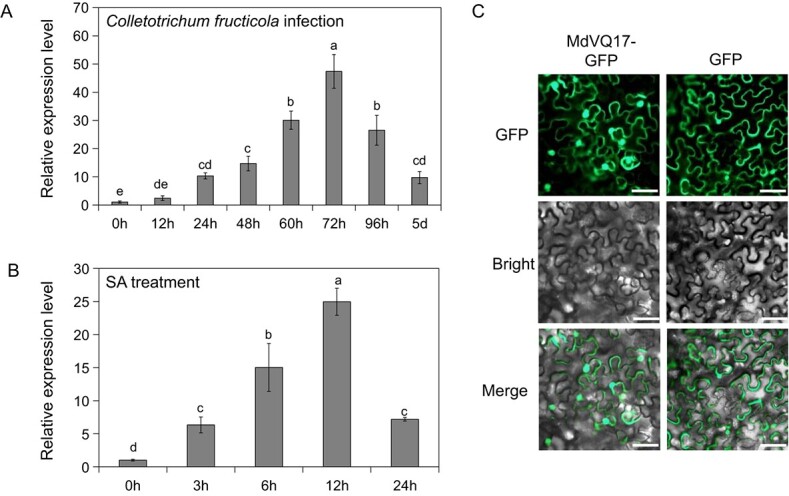
Expression patterns and subcellular localization of MdVQ17. **A**, **B** Relative expression levels of *MdVQ17* after *C. fructicola* infection (**A**) and SA treatment (**B**). Expression levels of *MdVQ17* were calculated relative to control (0 h) with the 2^–ΔΔCT^ method. Error bars represent the standard deviation based on three biological replicates. Bars labeled with different letters indicate values that are significantly different at *P* < 0.05, based on one-way ANOVA and Duncan’s test. **C** Subcellular localization of MdVQ17-GFP in *N. benthamiana* leaves. Scale bars = 50 μm.

In our previous studies, we identified VQ family genes from apple and cloned 28 of them [15]. Here, we analyzed the expression levels of the cloned *MdVQs* after *C. fructicola* infection via reverse transcription–quantitative polymerase chain reaction (RT–qPCR). The expression levels of most *MdVQs* were significantly upregulated by *C. fructicola* inoculation, whereas several *MdVQs* were downregulated (Supplementary Data Fig. S1), indicating that multiple *MdVQ* genes may be involved in the response to GLS infection in apples. Six *MdVQ* genes showed significant response to *C. fructicola* infection (fold change >10), among which *MdVQ17* exhibited the most significant change in gene expression (Supplementary Data Fig. S1). Therefore, *MdVQ17* was chosen for subsequent genetic transformation and functional identification in apple. The coding sequence (CDS) was 486 bp in length and encoded a protein with 161 amino acids. Phylogenetic analysis suggested that MdVQ17 was on the same branch as AtVQ12 and AtVQ29 in *Arabidopsis* (Supplementary Data Fig. S2A). Sequence alignment indicated that MdVQ17 and AtVQ12 had the highest similarity, although the amino acid sequence identity was only 22.36% (Supplementary Data Fig. S2B). Expression analysis showed that *MdVQ17* expression was significantly upregulated after 24 h of inoculation compared with 0 h, and continued to increase with time, peaked at 72 h, and then gradually decreased (Fig. 1A). Similarly, *MdVQ17* expression was notably induced at 3 h after SA treatment, reached a peak at 12 h, and then declined (Fig. 1B). Subcellular localization results for tobacco leaves indicated that MdVQ17 was distributed throughout the cell, similar to the GFP control (Fig. 1C).

### *MdVQ17* negatively regulates resistance to Glomerella leaf spot in apple

**Figure 2:**
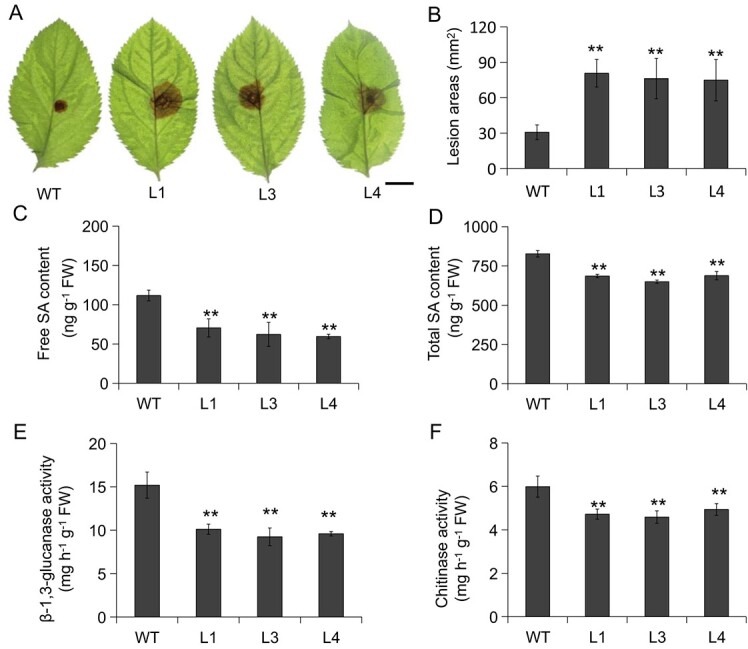
*MdVQ17*-OE increases *C. fructicola* susceptibility and inhibits SA accumulation in apple leaves. **A**, **B** Phenotypes (**A**) and lesion areas (**B**) of detached leaves of WT and *MdVQ17*-OE transgenic lines (L1, L3, and L4) after *C. fructicola* infection. Leaf samples were photographed 5 days after inoculation. Scale bar = 1 cm. Error bars represent the standard error (*n* = 18). Three experiments were repeated with similar results. Results of a typical experiment are shown. **C**, **D** Content of endogenous free SA (**C**) and total SA (**D**) in detached leaves of WT and *MdVQ17*-OE lines after inoculation with *C. fructicola*. **E**, **F** Activities of β-1,3-glucanase (**E**) and chitinase (**F**) in detached leaves of WT and *MdVQ17*-OE lines after *C. fructicola* infection. In **C**–**F**, error bars represent the standard deviation based on three biological replicates. **Significantly different relative to control at *P* < 0.01 according to Student’s *t-*test.

To investigate the role of MdVQ17 in the GLS infection response, several *MdVQ17*-overexpression (OE) transgenic apple lines with high *MdVQ17* expression (L1, L3, and L4) were generated (Supplementary Data Fig. S3). Five days after *C. fructicola* inoculation, leaves of transgenic lines exhibited a hypersensitive phenotype to *C. fructicola* compared with wild-type (WT) plants; the lesion area in *MdVQ17*-OE lines was significantly larger than in WT (Fig. 2A and B). Following *C. fructicola* inoculation, free SA and total SA contents in *MdVQ17*-OE transgenic lines were significantly lower than in WT plants (Fig. 2C and D). Moreover, the chitinase and β-1,3-glucanase activities in *MdVQ17*-OE transgenic lines were also significantly lower than in WT plants (Fig. 2E and F). These results indicate that *MdVQ17* overexpression increased the susceptibility of apple to GLS. To further investigate the role of MdVQ17 in the response to GLS, *MdVQ17*-RNAi (RNA interference) transgenic leaves were obtained using the transient transformation method (Supplementary Data Fig. S4A). However, after 3 days of *C. fructicola* inoculation, no significant differences in lesion size and related enzyme activities were observed between *MdVQ17*-RNAi and control (empty vector [EV]-RNAi) lines (Supplementary Data Fig. S4B–E).

### MdWRKY17 interacts with MdVQ17 *in vitro* and *in vivo*

**Figure 3:**
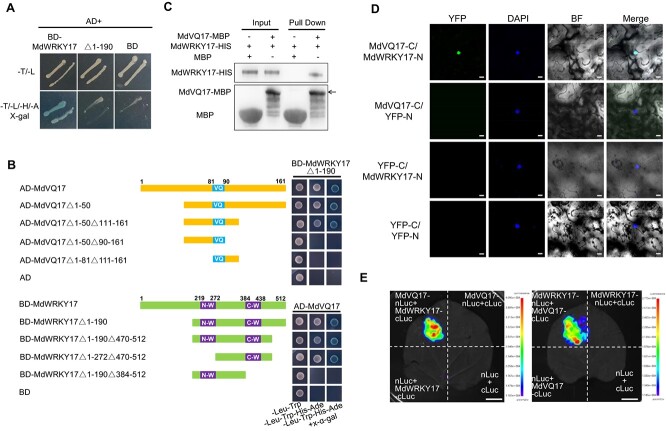
MdVQ17 physically interacts with MdWRKY17 *in vitro* and *in vivo*. **A** Identification of self-activation activity of full-length and truncated MdWRKY17 (MdWRKY17Δ1–190) based on Y2H assays. AD, pGAD424 prey vector; BD, pGBT9 bait vector; T, Trp; L, Leu; H, His; A, Ade. **B** Determination of the primary regions for protein interactions between MdVQ17 and MdWRKY17 through Y2H assays. VQ, VQ motif of MdVQ17; N-WRKY (N-W) and C-WRKY (C-W), the two WRKY domains of MdWRKY17. **C**, **D** Identification of interaction between MdVQ17 and MdWRKY17 by pull-down (**C**) and BiFC (**D**) assays. DAPI was used to indicate the nucleus. Scale bars = 10 μm. **E** Split-LUC assays showing MdVQ17–MdWRKY17 interaction in *N. benthamiana* leaves. In **D** and **E**, representative images are shown. Scale bars = 1 cm.

To explore how MdVQ17 regulates GLS resistance in apple, using MdVQ17-BD vector as a bait, we screened the apple cDNA library through a yeast two-hybrid (Y2H) system, then MdWRKY17 was identified as a possible MdVQ17-interacting protein. The full-length MdWRKY17 protein showed strong self-activation activity in yeast. Therefore, 190 amino acids in its N-terminal were deleted (MdWRKY17Δ1–190) to abolish this activity (Fig. 3A). As shown in Fig. 3B, the full-length MdVQ17 protein interacted with MdWRKY17Δ1–190 in yeast. To determine which parts of MdVQ17 and MdWRKY17 proteins are the primary regions for their interactions, four truncated forms of MdVQ17 (Δ1–50, Δ1–50Δ111–161, Δ1–50Δ90–161, and Δ1–81Δ111–161) and four truncated forms of MdWRKY17 (Δ1–190, Δ1–190Δ470–512, Δ1–272Δ470–512, and Δ1–190Δ384–512) were further generated and used for Y2H assays in different combinations. Deletion of the N- and C-terminus of MdVQ17 or the N-terminus of MdWRKY17, which contains a WRKY domain (N-WRKY), did not affect MdVQ17–MdWRKY17 interactions. However, deleting the flanking sequence of the VQ domain of MdVQ17 or the C-WRKY domain of MdWRKY17 eliminated their physical interactions (Fig. 3B). These results demonstrated that the VQ domain and its flanking region of MdVQ17 and the C-WRKY domain of MdWRKY17 play critically important roles in their interactions. To further demonstrate the MdVQ17–MdWRKY17 interaction, pull-down assays were conducted. MdWRKY17–HIS could be captured by MdVQ17–MBP, but not by MBP (Fig. 3C), which indicates that interactions between MdVQ17 and MdWRKY17 occurred *in vitro*.

Subsequently, bimolecular fluorescence complementation (BiFC) and split-luciferase (LUC) assays were performed to demonstrate the interactions *in vivo*. In BiFC assays, an obvious yellow fluorescent protein (YFP) signal could be detected in the nucleus when MdVQ17-C was co-infiltrated with MdWRKY17-N in tobacco leaves, and no fluorescence was detected in control combinations containing YFP-C or YFP-N empty vectors (Fig. 3D). In split-LUC assays, strong LUC activity was observed when combinations MdVQ17-nLuc + MdWRKY17-cLuc or MdVQ17-cLuc + MdWRKY17-nLuc were co-expressed in tobacco leaves (Fig. 3E). These fluorescence observation results suggested that MdVQ17 interacts with MdWRKY17 *in vivo*.

### MdVQ17–MdWRKY17 interaction promotes the transcriptional activation and binding activity of MdWRKY17 on the *MdDMR6* promoter

**Figure 4:**
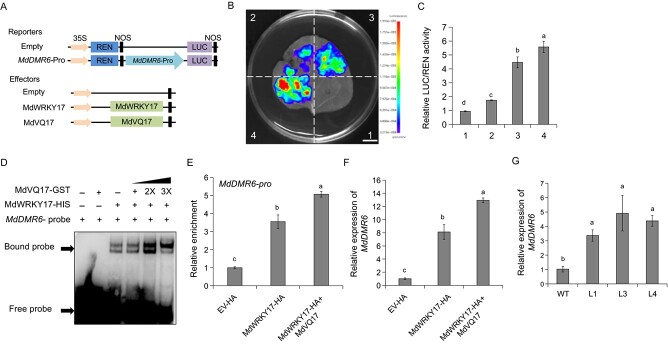
MdVQ17 promotes the binding and transcriptional activation activity of MdWRKY17 on the *MdDMR6* promoter. **A** Schematic diagrams of the constructed reporter and effector vectors used in transient expression assays. **B**, **C** Fluorescence (**B**) and relative LUC/REN activity (**C**) indicating the positive effect of MdVQ17 on MdWRKY17-activated transcription of the *MdDMR6* promoter. 1, empty effector + empty reporter; 2, empty effector + *proMdDMR6*::LUC; 3, 35S::MdWRKY17 + *proMdDMR6*::LUC; 4, 35S::MdVQ17 + 35S::MdWRKY17 + *proMdDMR6*::LUC. Empty reporter and effector vectors were used as controls. Scale bar = 1 cm. **D** EMSAs showing that MdVQ17 promoted the binding of MdWRKY17 protein to the *proMdDMR6*. 2× and 3× represent the multiples of the MdVQ17-GST protein. **E** ChIP–qPCR assays showing that MdVQ17 promoted the binding of MdWRKY17 to *proMdDMR6* in apple. Relative enrichment was calculated as the ratio of *MdWRKY17/MdVQ17* transgenic lines to control (EV-HA). **F** Expression analysis of *MdDMR6* in *MdWRKY17* and *MdVQ17* transgenic leaves. Leaves treated with spore suspension for 12 h were harvested for RT–qPCR analysis. **G** Expression level of *MdDMR6* in *MdVQ17*-OE lines. Error bars indicate the standard deviation of three biological replicates in all bar graphs. Different letters represent significant differences based on one-way ANOVA and Duncan’s tests (*P* < 0.05).

A previous study discovered that MdWRKY17 directly upregulated *MdDMR6* gene expression to promote SA degradation, thereby negatively regulating GLS resistance in apple [3]. To clarify the effect of MdVQ17 on MdWRKY17 transcriptional activity, dual-LUC assays were conducted. The sequence of the *MdDMR6* promoter was inserted into the pGreenII 0800-LUC vector, and *MdWRKY17* and *MdVQ17* CDSs were inserted into the pGreenII 62-SK vector (Fig. 4A). The relative LUC/REN (firefly luciferase/*Renilla* luciferase) activity and fluorescence intensity were significantly higher when MdWRKY17 was expressed compared with the *MdDMR6pro*::LUC + empty effector vector combination. When co-expressing MdVQ17 in combination, there was a notable enhancement in MdWRKY17-mediated transcriptional activation on the *MdDMR6* promoter (Fig. 4B and C).

To determine if MdVQ17 affects the promoter-binding ability of MdWRKY17, probes were synthesized based on the *MdDMR6* promoter sequence, and electromobility shift assays (EMSAs) were conducted. Clear bands could be observed when MdWRKY17 was incubated with the *MdDMR6* probe, and the bands became deeper as the MdVQ17 protein content increased (Fig. 4D), indicating that MdVQ17 enhanced the binding ability of MdWRKY17 to the *MdDMR6* promoter. To further verify this result *in vivo*, the *MdWRKY17*-HA vector was constructed and transiently expressed in the leaves of GL-3 and *MdVQ17*-OE plants (Supplementary Data Fig. S5). These transgenic leaves were then used for chromatin immunoprecipitation (ChIP)–qPCR analysis after 12 h of *C. fructicola* inoculation. The results showed that MdVQ17 notably enhanced the binding activity of MdWRKY17 to the *MdDMR6* promoter (Fig. 4E). We also detected *MdDMR6* expression in these transgenic leaves. Following *C. fructicola* inoculation for. 12 h, *MdWRKY17*-OE notably promoted *MdDMR6* expression, which was further enhanced by *MdVQ17* co-expression (Fig. 4F). We detected *MdDMR6* expression levels in *MdVQ17*-OE transgenic lines and found that transgenic lines expressed significantly higher expression levels of *MdDMR6* than WT (Fig. 4G). These results indicated that MdVQ17 has a positive effect on the transcriptional activation and binding activity of MdWRKY17 on the *MdDMR6* promoter.

### DNA affinity purification sequencing analysis revealed that *MdPL-like* was a direct target of MdWRKY17

**Figure 5:**
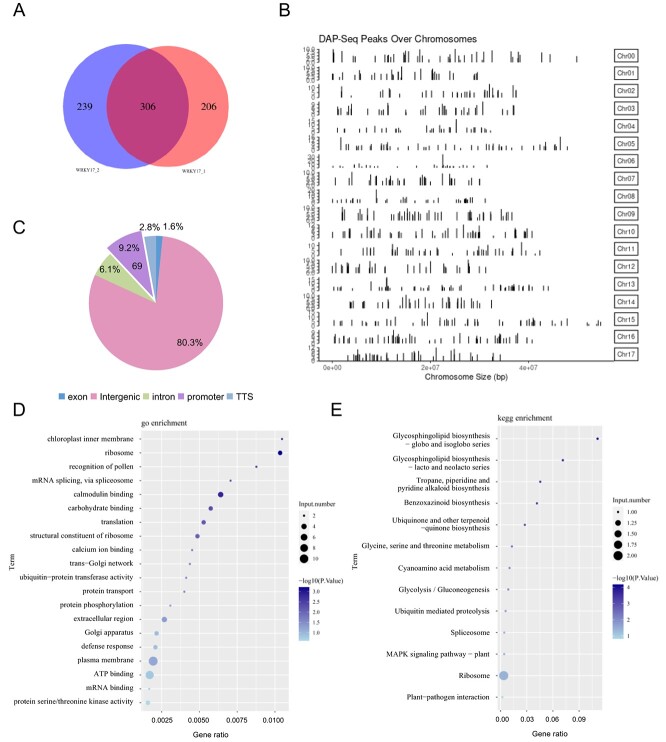
DAP-seq identified several potential target genes of MdWRKY17 in apple at the genome-wide level. **A** Venn diagrams of MdWRKY17 binding peaks. WRKY17-1 and WRKY17-2 are two technical replicate experiments. **B** Diagram of the distribution of identified peaks in different apple chromosomes. **C** Statistical analysis of the distribution of identified peaks in different functional regions of the genome. **D**, **E** GO enrichment (**D**) and KEGG pathway enrichment (**E**) analyses of candidate target genes of MdWRKY17.

DNA affinity purification sequencing (DAP-seq) was performed to explore the potential target genes of MdWRKY17 at the genome-wide level (Fig. 5). A total of 751 potential binding sites were obtained using two technical replicates (Fig. 5A), which were widely distributed in 18 chromosomes of apple (Fig. 5B). A total of 9.2% of these binding sites were located in the promoter region of 69 genes (Fig. 5C), which might be direct target genes of MdWRKY17. Gene Ontology (GO) and Kyoto Encyclopedia of Genes and Genomes (KEGG) pathway enrichment analyses showed that these genes are involved in various biological processes, including Ca^2+^ signaling, mitogen-activated protein kinase (MAPK) signaling, structural composition, protein transport, protein phosphorylation and ubiquitination, amino acid metabolism, mRNA splicing and translation, defense response, and plant–pathogen interaction (Fig. 5D and E).

**Figure 6:**
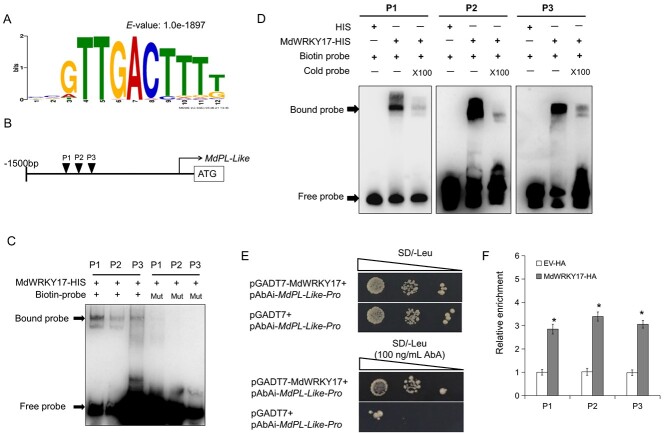
MdWRKY17 directly binds to the *MdPL-like* promoter. **A** Core DNA sequence of the most enriched MdWRKY17-binding motif in the DAP-seq results. **B** Diagram of the potential MdWRKY17-binding sites within the *MdPL-like* promoter. P1–P3 indicate the location of the three potential binding sites. **C** EMSAs showing the binding of MdWRKY17 to the *MdPL-like* promoter *in vitro*. **D** Identification of MdWRKY17-binding specificity to sites P1–P3 by competing EMSAs. ×100, multiple of the cold probe (without biotin labeling). **E** Y1H assays showing the interaction between MdWRKY17 and *MdPL-like* promoter. AbA, aureobasidin A. **F** ChIP–qPCR assays showing binding of MdWRKY17 to the *MdPL-like* promoter *in vivo*. Relative enrichment was calculated as the ratio of *MdWRKY17*-HA transgenic to control (EV-HA). Error bars represent the standard deviation based on three biological replicates. ^*^Significantly different relative to WT at *P* < 0.05 according to Student’s *t-*test.

Analysis using the Multiple EM for Motif Elicitation (MEME) suite revealed that the motif TTGACT, identified as a typical W-box element, exhibited the highest enrichment of MdWRKY17 binding sites (Fig. 6A). By screening functional annotation of MdWRKY17 potential target genes, *MdPL-like*, a gene belonging to the pectin lyase-like superfamily (Supplementary Data Fig. S6), was identified and selected for subsequent analysis. The RT–qPCR results showed that the expression of *MdPL-like* was upregulated by *C. fructicola* infection (Supplementary Data Fig. S7), suggesting that it potentially plays an important role in the apple’s defense mechanism against *C. fructicola*. To verify whether *MdPL-like* is a direct target of MdWRKY17, EMSA, yeast one-hybrid (Y1H), and ChIP–qPCR assays were carried out. Promoter sequence analysis revealed the presence of three potential W-box binding sites (P1–P3) within the *MdPL-like* promoter (Fig. 6B). Subsequently, EMSA results indicated that MdWRKY17 could directly bind to all three of these sites, and competitive EMSAs confirmed the binding specificity by the addition of mutant and competing probes (Fig. 6C and D). The binding of MdWRKY17 to the *MdPL-like* promoter was also confirmed in yeast through Y1H assays. Yeast cells transformed with pGADT7-MdWRKY17 and pAbAi-*MdPL-like*-Pro could grow on SD/−Leu medium with 100 ng/ml aureobasidin A (AbA); however, yeast cells transformed with pGADT7 empty vector and pAbAi-*MdPL-like*-Pro did not survive (Fig. 6E). Subsequently, ChIP–qPCR assays were conducted to demonstrate this binding relationship *in vivo*, and the results suggested that MdWRKY17 bound to the three putative binding sites P1–P3 in the *MdPL-like* promoter *in vivo* (Fig. 6F). These results indicated that *MdPL-like* was a direct downstream target of MdWRKY17.

### MdVQ17 promotes the transcriptional activation and binding activity of MdWRKY17 on the *MdPL-like* promoter

**Figure 7:**
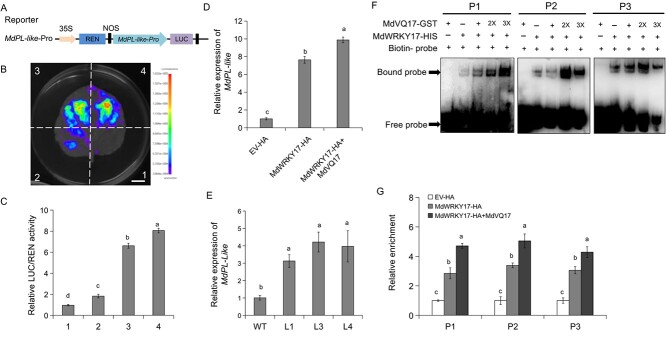
MdVQ17 promotes the binding and transcriptional activation activity of MdWRKY17 on the *MdPL-like* promoter. **A** Schematic diagram of the reporter vector. **B**, **C** Fluorescence (**B**) and relative LUC/REN activity (**C**) showing that co-expression of MdVQ17 enhanced MdWRKY17-activated transcription of the *MdPL-like* promoter. 1, empty effector + empty reporter; 2, empty effector + *proMdPL-like*::LUC; 3, 35S::MdWRKY17 + *proMdPL-like*::LUC; 4, 35S::MdVQ17 + 35S::MdWRKY17 + *proMdPL*-like::LUC. Scale bar = 1 cm. **D** Expression analysis of *MdPL-like* in *MdWRKY17* and *MdVQ17* transgenic leaves. Apple leaves treated with spore suspension for 12 h were harvested for RT–qPCR analysis. **E** Expression levels of *MdPL-like* in *MdVQ17*-OE leaves. **F** EMSAs showing that MdVQ17 enhances the promoter-binding ability of MdWRKY17. **G** ChIP–qPCR assays showing that MdVQ17 promotes the binding of MdWRKY17 to the *MdPL-like* promoters in apple. In **D**, **E**, and **G**, error bars represent the standard deviation based on three biological replicates. Different letters in bar graphs indicate significant differences between treatments, according to one-way ANOVA and Duncan’s test (*P* < 0.05).

Having demonstrated the MdVQ17–MdWRKY17 interaction (Fig. 3), further investigations were conducted to understand whether MdVQ17 could affect the transcriptional regulatory activity of MdWRKY17 on *MdPL-like*. The promoter sequence of *MdPL-like* was inserted into the pGreenII 0800-LUC vector (Fig. 7A), and dual-LUC assays were performed. Co-expression of the MdWRKY17 protein significantly increased the relative LUC/REN activity and fluorescence intensity compared with the control combinations containing empty effector vector (Fig. 7B and C), indicating that MdWRKY17 activated the transcription of *MdPL-like*. The co-expression of MdVQ17 and MdWRKY17 further enhanced relative LUC/REN activity and fluorescence intensity (Fig. 7B and C), suggesting that MdVQ17 positively regulates MdWRKY17-mediated *MdPL-like* transcription. To further identify the role of MdWRKY17 and MdVQ17 in *MdPL-like* gene expression, we determined its expression in *MdWRKY17* and *MdVQ17* transgenic apple leaves through RT–qPCR. The expression of the *MdPL-like* gene was markedly higher in *MdWRKY17*-HA transgenic leaves compared with the control (EV-HA), and its expression was further promoted by *MdVQ17* co-expression (Fig. 7D). Moreover, *MdPL-like* expression in *MdVQ17*-OE leaves was significantly higher than in WT leaves (Fig. 7E).

To investigate the effect of MdVQ17 on the binding ability of MdWRKY17 to the *MdPL-like* promoter, EMSAs were conducted. The addition of MdVQ17 protein could significantly enhance the binding of MdWRKY17 to the *MdPL-like* promoter (Fig. 7F). ChIP–qPCR assays were then carried out to verify the positive effect of MdVQ17 on the MdWRKY17 promoter binding ability *in vivo*. *MdVQ17*-OE notably enhanced the binding activity of MdWRKY17 on the *MdPL-like* promoter 12 h after *C. fructicola* inoculation (Fig. 7G). The above results suggested that MdVQ17 functions positively in promoting the binding and transcriptional activation activity of MdWRKY17 on the *MdPL-like* promoter.

### *MdPL-like* negatively regulates resistance to Glomerella leaf spot in apple

**Figure 8:**
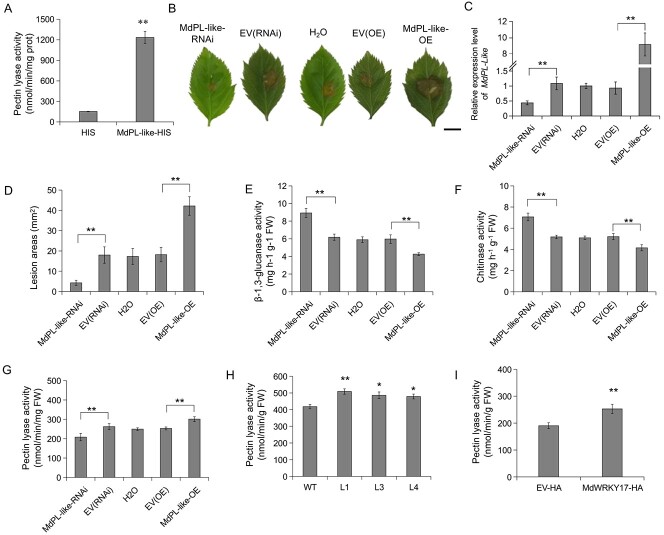
*MdPL-like* promotes pectin lyase activity and susceptibility to *C. fructicola* in apple leaves. **A** Pectin lyase activity of purified HIS and MdPL-like–HIS proteins. **B** Disease symptoms on infiltrated leaves of *MdPL-like*-OE and *MdPL-like*-RNAi plants after 3 days of *C. fructicola* infection. Scale bar = 0.5 cm. **C** Relative expression levels of *MdPL-like* in *MdPL-like* transgenic leaves. **D** Lesion areas of *MdPL-like* transgenic leaves after inoculation with *C. fructicola*. Error bars represent the standard error (*n* = 18). **E**–**G** Activities of β-1,3-glucanase (**E**), chitinase (**F**), and pectin lyase (**G**) in *MdPL-like* transgenic leaves after *C. fructicola* infection. **H** Pectin lyase activity in WT and *MdVQ17*-OE line (L1, L3, and L4) leaves after *C. fructicola* infection. **I** Pectin lyase activity in *MdWRKY17*-HA transgenic leaves after *C. fructicola* infection. In **C** and **E**–**I**, error bars represent the standard deviation based on three biological replicates. ^*^P < 0.05, ^**^P < 0.01: significantly different relative to WT according to Student’s *t-*test.

The phylogenetic tree indicated that *MdPL-like* is a pectin lyase-encoding gene (Supplementary Data Fig. S6). To determine whether the protein encoded by *MdPL-like* has pectin lyase activity, the purified MdPL-like–HIS fusion protein was obtained, and the catalytic activity of the purified protein was measured. Compared with the HIS protein control, the MdPL-like–HIS fusion protein exhibited high pectin lyase activity (Fig. 8A), indicating that the protein encoded by *MdPL-like* has pectin lyase activity.

To investigate the role of *MdPL-like* in modulating GLS resistance in apple, *MdPL-like*-OE and *MdPL-like*-RNAi vectors were constructed and transiently expressed in apple leaves (Fig. 8B and C). After 3 days of inoculation with *C. fructicola*, the lesion area in *MdPL-like*-OE and *MdPL-like*-RNAi transgenic leaves was significantly larger and smaller than that of the controls (EV-OE/EV-RNAi), respectively (Fig. 8B and D). Moreover, *MdPL-like* overexpression resulted in a significant decrease in chitinase and β-1,3-glucanase activities and increased pectin lyase activity in transgenic leaves, whereas the opposite pattern was observed for leaves in which *MdPL-like* expression was interfered with (Fig. 8E–G). These results indicated that *MdPL-like* plays a negative role in regulating apple GLS tolerance. Since both MdVQ17 and MdWRKY17 promote *MdPL-like* expression (Fig. 7), we examined the effects of these proteins on pectin lyase activity in transgenic apple leaves after *C. fructicola* infection. The pectin lyase activity in *MdVQ17*-OE and *MdWRKY17*-HA transgenic leaves was notably higher than that in the control (Fig. 8H and I). These results indicate that the MdVQ17–MdWRKY17 complex promotes pectin lyase activity by directly upregulating *MdPL-like* expression, thereby increasing the susceptibility of apple to *C. fructicola*.

## Discussion

GLS is one of the main diseases affecting apple yield in China. Some VQ genes have been reported to be involved in regulating plant resistance to pathogen infection in *Arabidopsis*, rice, and tomato [4, 14, 22]. For example, VQ10, VQ12, VQ16 (SIB2), VQ22 (JAV1), VQ23 (SIB1), and VQ29 from *Arabidopsis* and SlVQ15 from tomato participated in the response to the necrotrophic pathogen *B. cinerea* [4, 14]. VQ14 and VQ32 from rice play positive roles in regulating defense against bacterial blight [22]. However, there are few studies on the role of VQ proteins in regulating the response to pathogen infection in apple, especially GLS infection.

In our study, *MdVQ17* expression was highly upregulated by *C. fructicola* infection and exogenous SA treatment (Fig. 1A and B), indicating that *MdVQ17* plays a crucial role in the defense response of apple against GLS infection. Therefore, its role in regulating GLS tolerance in apple was clarified in detail through phenotype comparisons and determination of physiological indexes under *C. fructicola* infection. The accumulation of SA can promote the production of pathogenesis-related proteins [34]. Pathogenesis-related proteins generally have anti-proteolytic enzyme activity, and some also have chitinase or β-1,3-glucanase activity; they participate in the responses of plants to pathogens [35]. This study found that the SA levels and chitinase and β-1,3-glucanase activities were notably reduced in the *MdVQ17*-OE lines compared to the WT, leading to serious disease symptoms (Fig. 2). These results show that MdVQ17 negatively regulates the tolerance of apple to GLS, which might be related to its inhibition of SA accumulation. Unexpectedly, transgenic leaves in which *MdVQ17* expression was interfered with showed no difference in lesion size and related enzyme activity compared with the control (Supplementary Data Fig. S4). Given the large number of apple VQ family members [15], we suspect that this result might be related to the functional redundancy between MdVQ17 and other MdVQs. A similar finding has been made in many studies of multi-member families in plants. For example, in *Arabidopsis* the sensitivity of *vq18 vq26* double mutant seeds to ABA is higher than that of WT seeds. However, no conspicuous difference was observed between single-mutant *vq18*/*vq26* seeds and WT seeds [36]. Simultaneous silencing *Sl-EBF1* and *Sl-EBF2* led to a greater degree of curl in the leaves. In contrast, the growth phenotype of tomato plants with the *Sl-EBF1/2* gene silenced showed no significant difference compared with control plants [37]. The redundancy of genes ensures the stability of their functions by preventing them from being affected by individual gene mutations. The redundancy of genes also enhances the adaptability of organisms to different environments [38].

Multiple studies have shown that WRKY TFs can bind to W-box elements in the promoters of target genes, thereby changing the expression of these genes and participating in the plant’s response to pathogen infection [24, 25]. For instance, AtWRKY57 can bind to and activate the downstream target genes *jasmonate zim domain 1* (*JAZ1*) and *JAZ5*, thereby weakening resistance to *B. cinerea* [39]. OsWRKY51 in rice can bind to the W-box element of the *OsPR10a* promoter and positively regulate the defense response against *Xoo* by promoting the expression of *OsPR10a* [40]. VQ proteins are transcriptional regulators that can affect the transcriptional regulation of WRKY TFs on target genes through direct protein interactions [4]. For example, VQ20 interacts with WRKY2 and WRKY34, which enhances the transcriptional inhibitory activity of these proteins and regulates pollen development [41]. VQ9 interacts with WRKY8, which weakens the DNA-binding activity of WRKY8 and negatively regulates the WRKY8-mediated salt stress response [42]. The interaction between VQ16/VQ23 and WRKY57 affects the expression of *JAZ1*/*JAZ5*, and synergistically regulates the tolerance of *Arabidopsis* to *B. cinerea* [39]. In apple, MdWRKY15 and MdWRKY46 can bind to the promoters of the SA synthesis genes *MdICS1* and *MdPBS3.1*, respectively, and promote their expression, enhancing resistance to *Botryosphaeria dothidea* [43, 44]. By contrast, MdWRKY17 promotes SA degradation by directly promoting *MdDMR6* expression, and this has a negative regulatory effect on GLS tolerance in apples [3]. Here, MdWRKY17 was identified as an MdVQ17-interacting protein through Y2H assays, and the MdWRKY17–MdVQ17 interaction was further validated *in vivo* and *in vitro* (Fig. 3). These results, combined with the inhibitory effect of MdVQ17 on GLS tolerance and SA accumulation (Fig. 2), indicate that MdVQ17 might be involved in MdWRKY17-mediated SA degradation and GLS susceptibility through protein interactions. Therefore, the effect of MdVQ17 on promoter binding and the transcriptional activation activity of MdWRKY17 on the *MdDMR6* promoter was investigated using dual-LUC, EMSA, and ChIP–PCR assays. As expected, MdVQ17 co-expression significantly improved the binding and transcriptional activation activity of MdWRKY17 on the *MdDMR6* promoter (Fig. 4).

The positive role of MdVQ17 in promoting GLS susceptibility (Fig. 1) and *MdDMR6* expression (Fig. 4G) may lead to the question of whether MdVQ17 can directly bind to the promoter of the target gene and regulate their transcription independently of MdWRKY17. It has been suggested that VQ proteins function as transcriptional regulatory cofactors, not TFs. They generally do not bind directly to DNA, but typically work in tandem with various TFs through protein–protein interactions to fine-tune regulatory mechanisms in response to the external environment [4]. Therefore, recent studies of VQ proteins have not focused on their own promoter binding ability, but on their effects on the promoter binding and transcriptional activation activities of TFs with which they interact, such as VQ18 and VQ26 in *Arabidopsis* [36], SlVQ21, SlVQ16, and SlVQ15 in tomato [14, 45], and MdVQ10 in apple [28]. In this study, although the promoter binding ability of MdVQ17 was not studied in detail, the EMSAs at least showed that MdVQ17 cannot directly bind to the binding sites of MdWRKY17, but can promote the binding of MdWRKY17 on the *MdDMR6* promoter (Fig. 4D). These findings shed light on the molecular mechanism of the VQ family proteins involved in the response to pathogen infection in apple.

The first barrier against pathogen invasion is the cell wall. Many studies have shown that pathogens degrade plant cell walls by releasing pectin lyase to facilitate their invasion [46, 47]. Some recent studies have shown that plant pectin lyases mediate interactions between plants and pathogens. The pectin content is increased in *pmr5* and *pmr6* mutants, and these mutants show enhanced resistance to powdery mildew in *Arabidopsis* [30, 48]. During bacterial spot infection, bHLH3 and bHLH6 activate the expression of the pectin lyase gene *Solyc05g014000* to induce water-soaked disease lesions on tomato leaves [49]. In addition, RNAi of the pectate lyase-encoding gene *SlPL* increases resistance to grey mold in tomato [50]. In our study, we further probed the molecular mechanism underlying the susceptibility to GLS mediated by the MdWRKY17–MdVQ17 complex by performing DAP-seq to identify potential target genes (Fig. 5). *MdPL-like* was identified to be a direct target of MdWRKY17, and the binding of MdWRKY17 to the *MdPL-like* promoter was verified through Y1H, ChIP–qPCR, and EMSAs (Fig. 6). Moreover, MdVQ17 promoted the binding and transcriptional activation activity of MdWRKY17 on the *MdPL-like* promoter (Fig. 7), and the effect of *MdPL-like* in promoting pectin lyase activity and GLS susceptibility in apple was characterized in detail (Fig. 8). We noted that *MdPL-like*-OE significantly inhibited chitinase and β-1,3-glucanase activities in apple leaves after *C. fructicola* infection (Fig. 8E and F). In tobacco and *Arabidopsis*, transgenic plants expressing an attenuated version of *endo*-polygalacturonase (PG) of *Aspergillus niger* showed enhanced resistance to fungal infection, accompanied by increased peroxidase and β-1,3-glucanase activities [51]. It has been reported that the accumulation of SA affects chitinase and β-1,3-glucanase activities [34, 35]. Moreover, the SA-mediated signaling pathway activated by bacteria can be induced by treatment with pectate lyases (Pels: PelB, PelI, and PelL), whereas the JA- and ethylene-mediated signaling pathways respond only to PelB and PelI [52]. These results suggest that the influence of *MdPL-like* on chitinase and β-1,3-glucanase activities may be related to the SA signaling pathway. Further research is necessary to clarify the role of *MdPL-like* in SA accumulation and signaling in apple. Overall, our results indicate that, in addition to promoting SA degradation by regulating *MdDMR6* expression, the MdWRKY17-MdVQ17 module can increase apple GLS susceptibility by directly promoting *MdPL-like* expression. These findings contribute to our knowledge of the diverse mechanisms involved in the immune response to pathogen invasion mediated by the WRKY–VQ complex.

**Figure 9:**
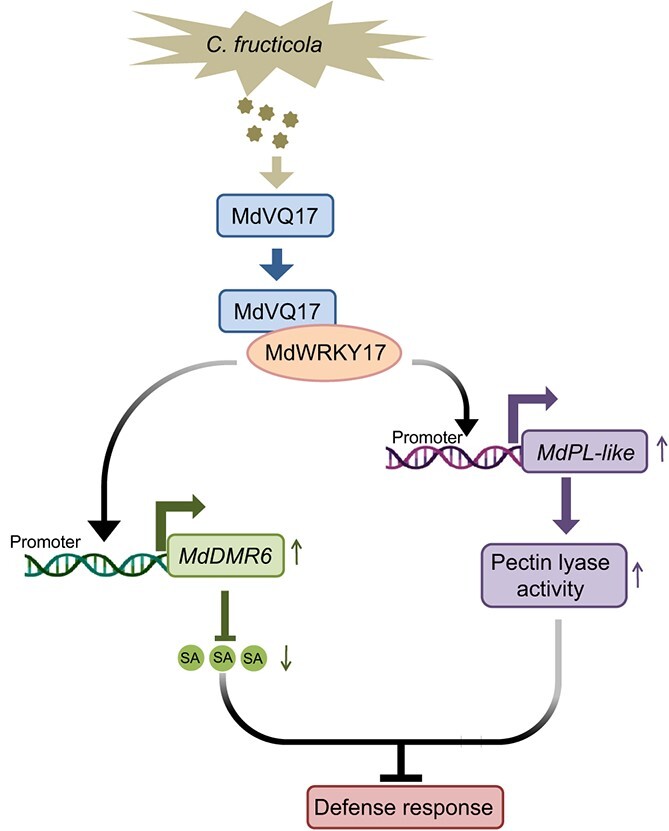
A functional model of MdVQ17 in response to GLS infection in apple. After the plant is infected with GLS, the expression of *MdWRKY17* and *MdVQ17* is activated, and the proteins they encode accumulate. MdVQ17 interacts with MdWRKY17 to enhance the binding and transcriptional activation activity of MdWRKY17 on the *MdDMR6* and *MdPL-like* promoters, thereby promoting MdWRKY17-mediated *MdDMR6* and *MdPL-like* expression and thus SA degradation and pectin lyase activity, resulting in increased GLS susceptibility in apple.

In sum, our findings demonstrated that MdVQ17 negatively regulates apple GLS tolerance by promoting MdWRKY17-mediated SA degradation and pectin lyase activity through the regulation of *MdDMR6* and *MdPL-like* expression. We propose a working model to describe the role of the MdVQ17–MdWRKY17 complex in response to GLS infection in apple (Fig. 9). Following GLS infection, the expression of *MdWRKY17* and *MdVQ17* is activated, and the proteins they encode accumulate. MdWRKY17 directly binds to the *MdDMR6* and *MdPL-like* promoters to activate their expression, thereby promoting SA degradation and pectin lyase activity, which results in reduced resistance to GLS. Concurrently, MdVQ17 interacts with MdWRKY17 to enhance its promoter binding and transcriptional activation activity, thereby increasing MdWRKY17-mediated GLS susceptibility. Our findings provide new insights that will aid future studies of the mechanisms underlying the regulation of stress responses mediated by the WRKY–VQ complex in plants. Additional studies are needed to clarify how *MdWRKY17* and *MdVQ17* are activated in response to GLS infection.

## Conclusion

In this study, MdVQ17 negatively regulates the tolerance of apple to GLS. We found that MdVQ17 can interact with MdWRKY17 to promote the binding and transcriptional activation activity of MdWRKY17 on the *MdDMR6* and *MdPL-like* promoters, thereby reducing the SA content and increasing pectin lyase activity, and increasing susceptibility to GLS. These results provide novel insights into the mechanism underlying the response to GLS infection in apple.

## Materials and methods

### Plant materials and stress treatments

GL-3 (a seedling clone from open-pollinated ‘Royal Gala’) apple plants were used for expression analysis and genetic transformation [53]. For expression analysis of *MdVQ17* and *MdPL-like* under *C. fructicola* infection, *C. fructicola* was inoculated on solid potato dextrose medium for 3–4 days at 25°C; a fungal tablet was then taken and cultured in liquid potato dextrose medium until the spore concentration was 10^6^ cfu ml^−1^ for fungal inoculation. The detached, fully expanded leaves of 60-day-old GL-3 plants were sprayed with the spore suspension and placed in a plastic box with moist filter paper, and the temperature was maintained at 25°C. Whole leaves were collected at 0, 12, 24, 48, 60, 72, and 96 h and 5 days after treatment for gene expression analysis. For expression analysis in response to SA, detached leaves of GL-3 plants were sprayed with 100 μM SA and incubated in the same way as described above. Whole leaves were collected at 0, 3, 6, 12 and 24 h post-treatment.

A 20 μl spore suspension was inoculated onto the center of detached leaves from *MdVQ17*-OE and WT plants as described previously [3]. On the 5th day of treatment, the detached leaves were collected for phenotypic comparison and stress-related indicator analysis. Additionally, a 10-μl spore suspension was inoculated onto the center of transiently OE or RNAi transgenic leaves in which *MdVQ17* and *MdPL-like* expression was overexpressed or inhibited. On the third day of treatment, the different transgenic leaves were collected for phenotypic comparison and stress-related indicator analysis. Three independent replications of the experiments were conducted, and 18 leaves from each line constituted a single biological replicate.

### Gene cloning and expression analysis

For cloning *MdVQ17*, *MdWRKY17*, and *MdPL-like*, their full-length CDSs were cloned from ‘Royal Gala’ apples leaves by RT–PCR.

The qRT–PCR experiments were performed using a LightCycler^®^ 96 instrument (Roche Diagnostics GmbH, Germany) along with SYBR Premix Ex Taq (Takara, Dalian, China). The 2^−ΔΔCT^ method was used to calculate expression values [54]. The *MdMDH* gene was used to normalize expression values [55]. The primers are listed in Supplementary Data Table S1.

### Vector construction and genetic transformation

The full-length CDS of *MdVQ17* was inserted into the overexpression pCambia2300 vector. For the genetic transformation of apple leaves we used *Agrobacterium*-mediated methods, introducing exogenous genes into GL-3 through strain EHA105 [53]. Following kanamycin screening, identification via PCR and qRT–PCR, stable genetic *MdVQ17-*OE transgenic apple lines were obtained.

Some transient transgenic plants were obtained. The *MdWRKY17* CDS was inserted into the overexpression pGWB415 vector (with a 3× HA tag fused in the N-terminal), and the *MdPL-like* CDS was inserted into the pCambia2300 vector. A specific 300-bp fragment of *MdVQ17* or *MdPL-like* was introduced into the RNAi vector pK7GWIWG2D. The primers are listed in Supplementary Data Table S1. These constructs were transiently transformed into the leaves of GL-3 or *MdVQ17*-OE plants using the EHA105-mediated method, as described previously [56]. Expression levels were determined 2 days after transient transformation; phenotypic comparisons and stress-related indicator measurements were performed after 3 days of infection. Three independent replications of the experiment were performed.

### Subcellular localization assays

The *MdVQ17* CDS was inserted into the pCambia2300-GFP vector, then expressed transiently in *Nicotiniana benthamiana* leaves through the *Agrobacterium* strain GV3101-mediated technique. After 3 days, an automatic fluorescence microscope (BX63, Olympus, Japan) was utilized to detect GFP fluorescence. The empty pCambia2300-GFP vector was used as control.

### Measurement of salicylic acid content and chitinase and β-1,3-glucanase activity

Leaf samples (0.1 g) from WT and *MdVQ17*-OE lines after inoculation with *C. fructicola* for 5 days were collected for SA content determination. SA was extracted and analyzed by liquid chromatography–mass spectrometry according to a previous method [16].

Chitinase and β-1,3-glucanase activities were measured using kits (JDZM-2-G and GA-2-Y; Comin, Suzhou, China).

### Pectin lyase activity determination

The pectin lyase activity in apple leaves was measured using a kit (PL-2-G; Comin). To determine the enzyme activity of MdPL-like *in vitro*, pET32a-*MdPL-like* (His tag) was constructed. The fusion protein MdPL-like–HIS was produced in *Escherichia coli* strain Rosetta and isolated using an HIS purification column (Beyotime, Shanghai, China). The MdPL-like–HIS protein was collected for pectin lyase activity assays. The concentrations of MdPL-like–HIS fusion protein were determined using the Bradford method [57].

### Yeast two-hybrid assays

The full-length CDSs and truncated fragments of *MdWRKY17*and *MdVQ17* were introduced into pGBT9 bait vector and pGAD424 prey vector, respectively. Utilizing the lithium acetate method, these constructs were co-transformed into yeast strain Y2H Gold with specified combinations and spread on synthetic dropout (SD) medium lacking Trp and Leu (SD/−Trp−Leu) and SD/−Trp−Leu−His−Ade (± X-α-gal) to confirm positive interactions. The empty pGBT9 or pGAD424 vectors were used as negative controls [58].

### Bimolecular fluorescence complementation assays

The *MdWRKY17* and *MdVQ17* CDSs were introduced into pSPYNE and pSPYCE vectors, respectively. *Agrobacterium* strain C58c1 was used to introduce these constructs into tobacco leaves in different combinations. Yellow fluorescence was observed through a confocal laser-scanning microscope (LSM510 META; Zeiss, Germany) after 2 days of infection. DAPI (4′,6-diamidino-2-phenylindole) was used for cell nucleus marking.

### Split-luciferase assays

The *MdWRKY17* and *MdVQ17* CDSs were inserted into pCAMBIA1300-nLuc or pCAMBIA1300-cLuc. EHA105 was used to introduce these constructs into tobacco leaves in different combinations. Three days later, spraying 1 mM fluorescein (Promega) on the leaves allowed the observation of fluorescence signal via a multispectral dynamic fluorescence microscopy imaging system (PlantView100, China).

### Pull-down assays

The pET32a-*MdWRKY17* (His tag) and pMAL-C5X-*MdVQ17* (MBP tag) recombinant vectors were created to express MdWRKY17–HIS and MdVQ17–MBP fusion proteins in *Escherichia coli* strain Rosetta. Following expression, the proteins were purified using HIS and MBP purification columns (Beyotime), respectively. Pull-down assays were conducted according to the method by Mao *et al*. [59]. The proteins were captured with anti-MBP amylose resin (New England Biolabs, Shanghai, China) for 8 h at 4°C. The eluted proteins were subsequently identified using western blot analysis with specific anti-HIS and -MBP antibodies (Beyotime). MBP protein was used as a negative control.

### DAP-seq analysis

The DAP-seq assays were conducted according to the method by Dong *et al*. [60]. ‘Gala’ leaves were used to extract genomic DNA (gDNA). Bluescape Scientific Co., Ltd (Hebei, China) handled the purification and sequencing of the DNA samples. Two technical replications were carried out. DAP-seq reads were aligned with the apple genome (GDDH13) (https://www.rosaceae.org/) utilizing Bowtie2 software [61]. MACS2 callpeak and IDR software were used to merge the peaks (*P* < 0.05) and score the reliability of peaks [62]. Analysis of conserved motifs within the peak regions and the annotation of bound peaks were performed using MEME-CHIP software and Homer software, respectively [63, 64].

### Yeast one-hybrid assays

The *MdWRKY17* CDS was integrated into the pGADT7 vector. A 1500-bp *MdPL-like* promoter was integrated into the pAbAi vector. They were simultaneously transformed into yeast strain Y1H and spread on SD medium lacking Leu with or without 100 ng/ml AbA to confirm positive interactions. The empty pGADT7 vector was used as the negative control.

### Electromobility shift assay assays

MdVQ17–GST and MdWRKY17–HIS were expressed and purified. Probes were designed according to the locations of the W-box element. Competitor probes (cold probes) were unlabeled probes. The probe sequences can be found in Supplementary Data Table S1. The Chemiluminescent EMSA Kit (Beyotime) was utilized for EMSAs.

### Chromatin immunoprecipitation–qPCR assays

*MdWRKY17*-HA transient transgenic apple leaves were treated with spore suspension for 12 h and then harvested and cross-linked by immersion in formaldehyde solution. After sonication, the chromatin was immunoprecipitated with or without anti-HA antibody. We used qPCR to determine the relative enrichment values of the promoter fragments, with the enrichment of control (EV-HA) used as the reference and set to 1.0. The experiment was replicated three times. Details of the primers used can be found in Supplementary Data Table S1.

### Dual-luciferase assays

The dual-LUC assays were conducted using the method by Yang *et al*. [65]. The *MdPL-like* and *MdDMR6* promoters (1500 bp in length) were individually inserted into pGreenII 0800-LUC reporter vectors. The *MdVQ17* and *MdWRKY17* CDSs were inserted into pGreenII 62-SK effector vectors. GV3101 was used to introduce these vectors into tobacco leaves in different combinations. After 2 days of incubation, LUC and REN activities were measured using a dual-LUC assay kit (Yeasen, Shanghai, China). Each combination underwent a minimum of six experimental replications.

### Statistical analysis

The data were analyzed using IBM SPSS 20.0. One-way analysis of variance (ANOVA) was utilized for comparing the differences between the control plants and transgenic lines. Duncan’s test or Student’s t-test was applied to assess the significance level.

## Supplementary Material

Web_Material_uhae159

## Data Availability

The sequencing information presented in this paper is available on the Apple Genome Database (https://iris.angers.inra.fr/gddh13/) as follows: MdVQ17 (MDP0000856686), MdWRKY17 (MD12G1181000), MdDMR6 (MD10G1053200), and MdPL-like (MD00G1117900). All data in this study are provided in the article and its supplementary materials.
