# Supplementary material for: MdVQ17 negatively regulates apple resistance to Glomerella leaf spot by promoting MdWRKY17-mediated salicylic acid degradation and pectin lyase activity
Source: Hortic Res. 2024 Jun 7;11(8):uhae159. doi: 10.1093/hr/uhae159 (PMC11298625; doi:10.1093/hr/uhae159)
Supplement: Web_Material_uhae159 [file web_material_uhae159.zip › supplementary materials.docx]

**Supporting information：**

**
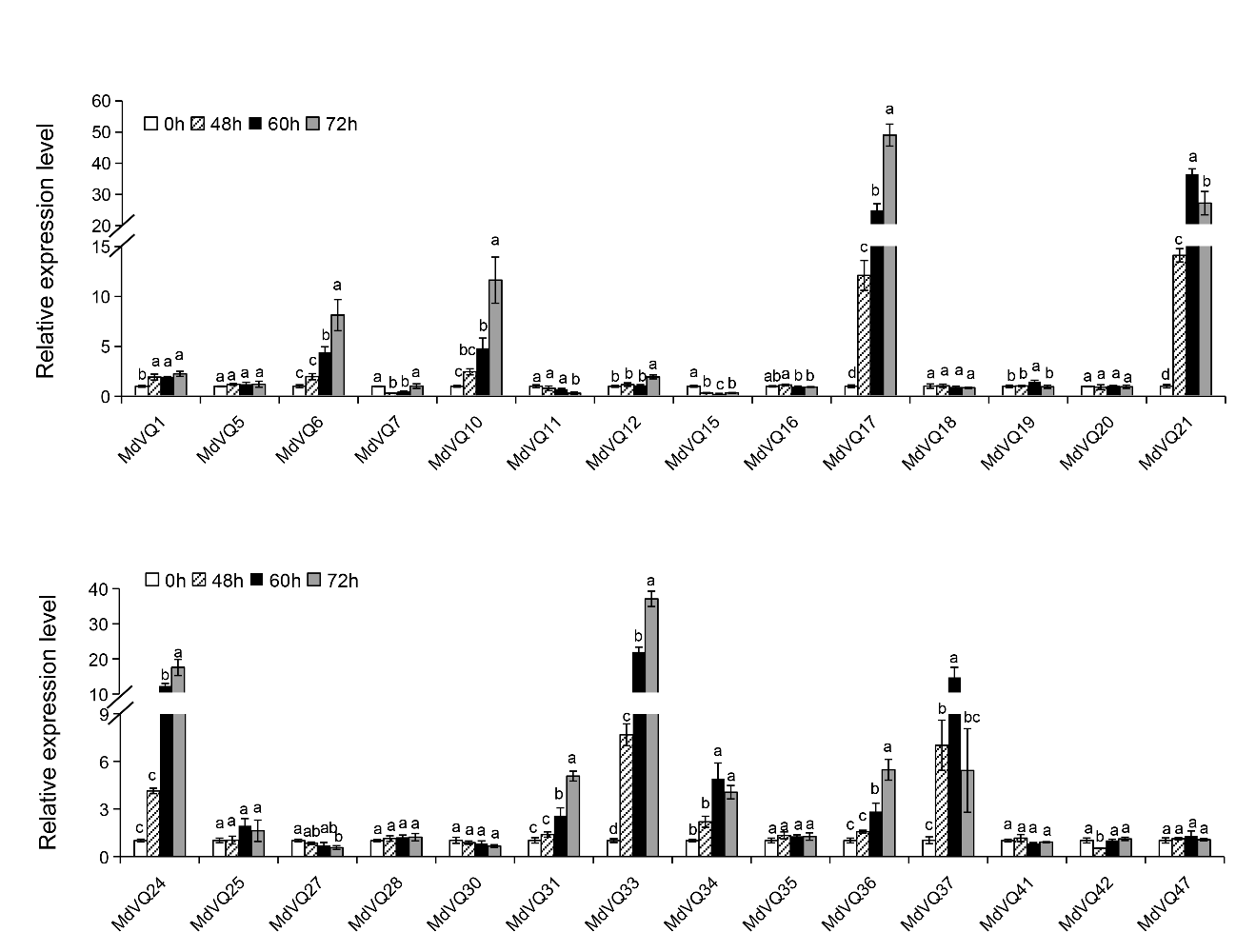
**

**Supplemental Figure 1**. **Expression pattern analysis of *MdVQs* genes after *C. fructicola* infection.** Relative expression values of each period compared to the control (0 h) were calculated; Error bars represent SD based on three biological replicates. Bars labeled with different letters indicate values that are significantly different at *P* <0.05, based on one-way ANOVA and Duncan’s test.


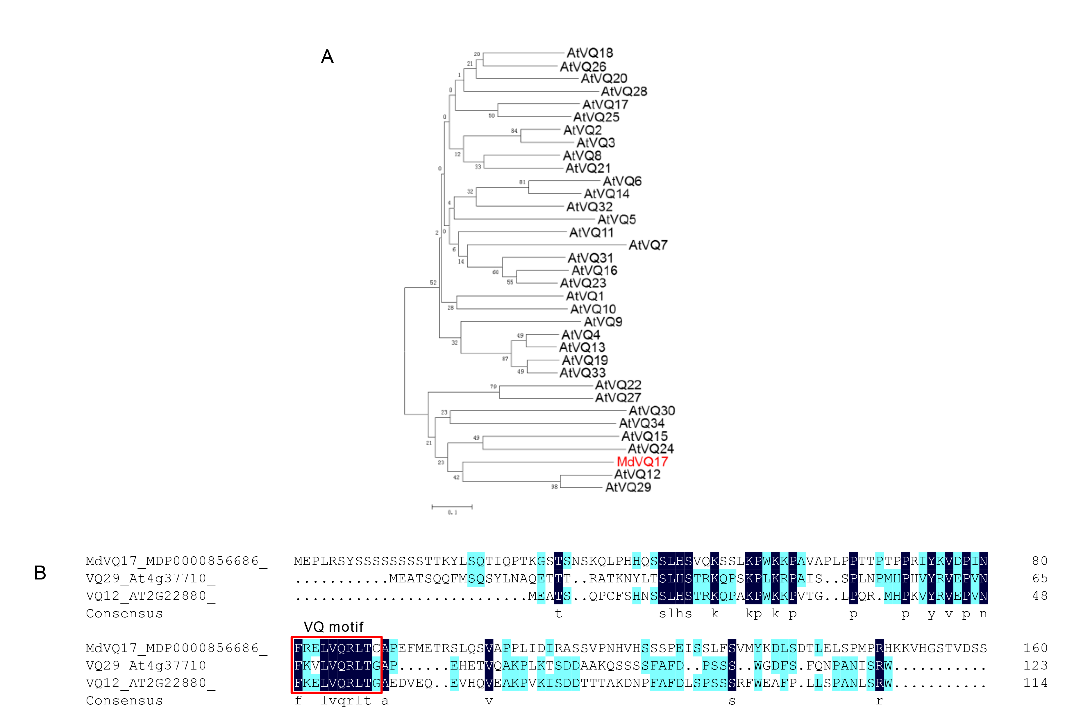


**Supplemental Figure 2**. **Protein sequence analysis of MdVQ17 and AtVQs.**

(A) Phylogenetic analysis of MdVQ17 and AtVQ proteins. (B) Sequence alignment of MdVQ17, AtVQ12, and AtVQ29 proteins. The red box indicates the conserved VQ motif.


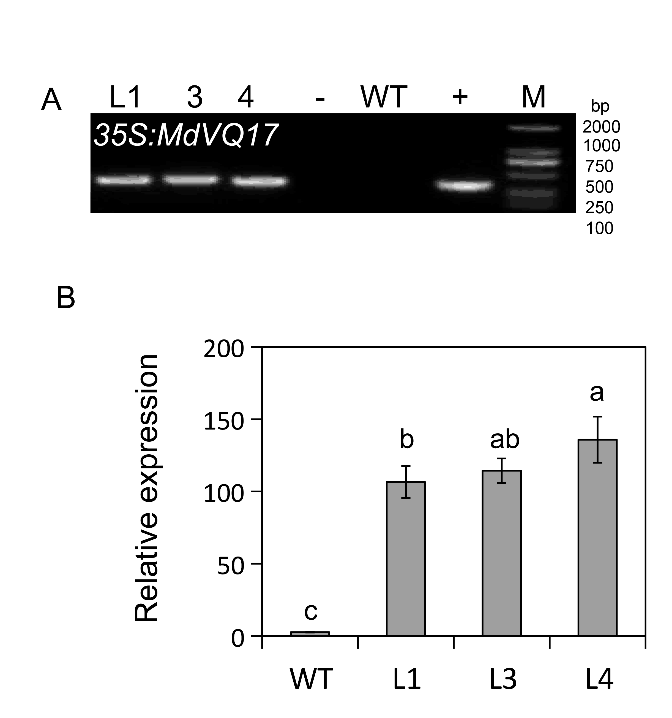

**Supplemental Figure 3**. **Identiﬁcation of *MdVQ17* transgenic apple plants.**

(A) Identiﬁcation of transgenes through genomic PCR. Genomic DNA was extracted from the leaves of apple plants. M, DNA marker; -, negative control (H_2_O); +, positive control, the *MdVQ17*-pCambia2300 plasmid. (B) Relative expression of *MdVQ17* in the leaves of WT and *MdVQ17*-OE transgenic plants.


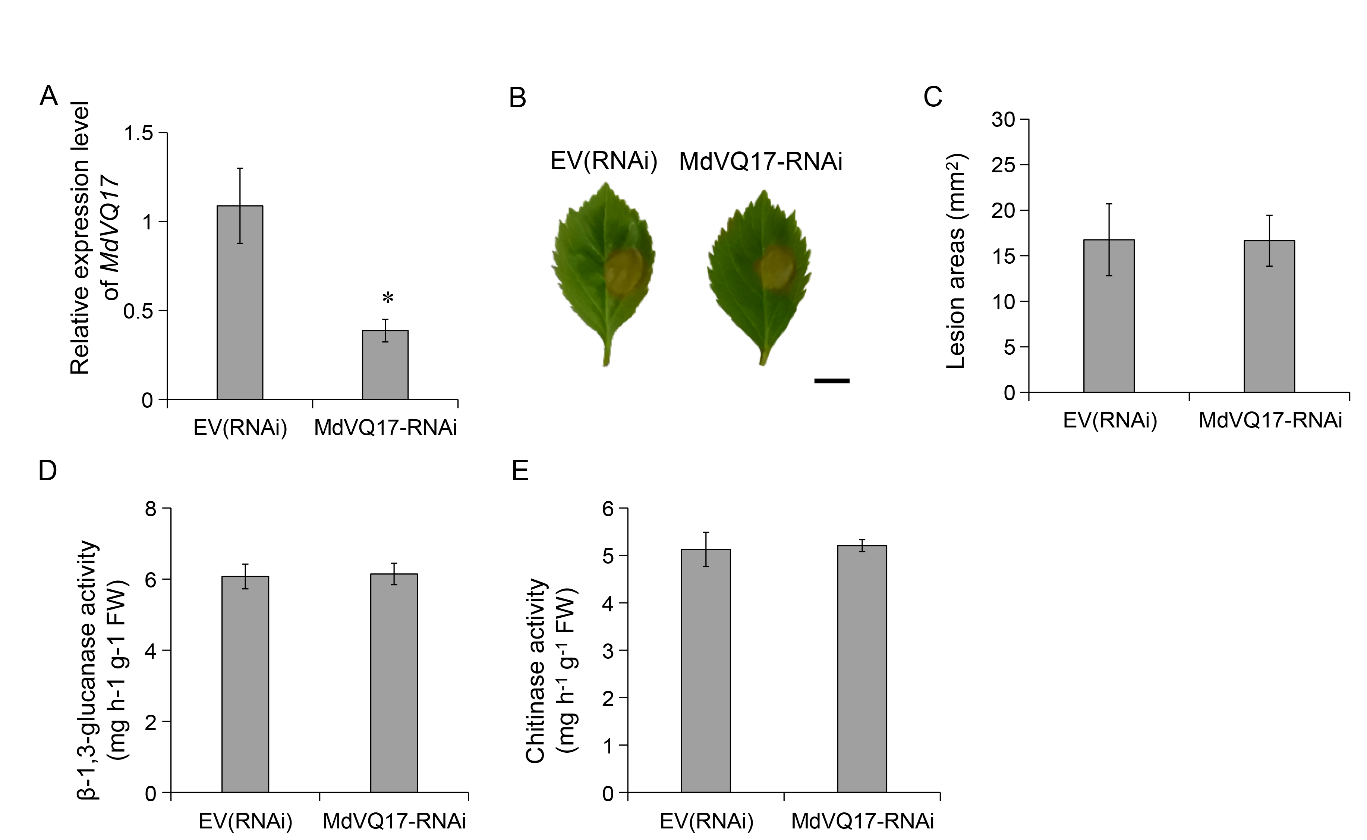


**Supplemental Figure 4**. **Interference with *MdVQ17* expression in apple leaves does not affect susceptibility to *C. fructicola*.**(A) Relative expression levels of *MdVQ17* in *MdVQ17*-RNAi transgenic leaves. Leaves transformed with empty pK7GWIWG2D vector (EV-RNAi) were used as controls. (B) Disease symptoms of *MdVQ17*-RNAi transgenic leaves after inoculation with *C. fructicola* for 3 days. Bars = 0.5 cm. (C) Lesion areas of the *MdVQ17*-RNAi transgenic leaves after inoculation with *C. fructicola*. Error bars represent SE (n = 18). The experiments were repeated three times with similar results. (D–E) The activities of β-1,3-glucanase (D) and chitinase (E) in apple leaves after inoculation with *C. fructicola*. Error bars in A, D, and E represent SD based on three biological replicates. * in each panel indicates values that are significantly different relative to the control at *P* < 0.05 according to Student’s *t-*test.


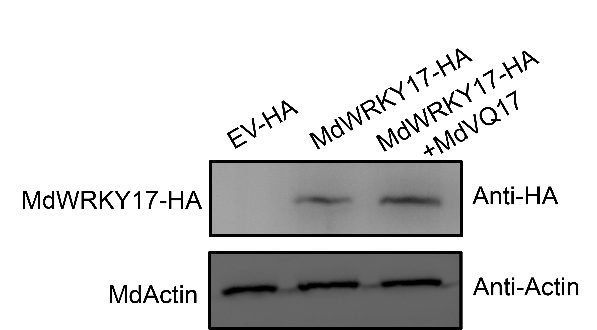

**Supplemental Figure 5**. **Identification of MdWRKY17-HA protein expression in transgenic leaves.** The protein levels of MdWRKY17-HA in different transgenic leaves were detected with anti-HA antibody through western blot.


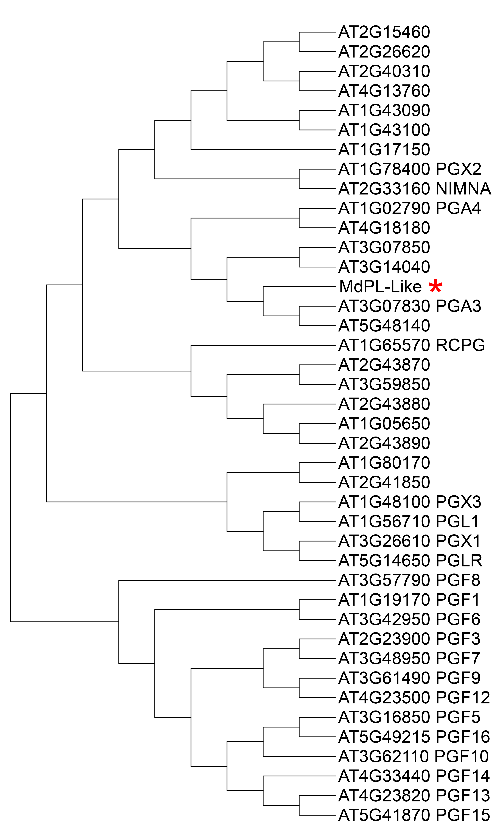

**Supplemental Figure 6.** **Phylogenetic analysis of MdPL-like and *Arabidopsis* pectin lyase-like superfamily proteins.** MdPL-like is marked by a red asterisk.


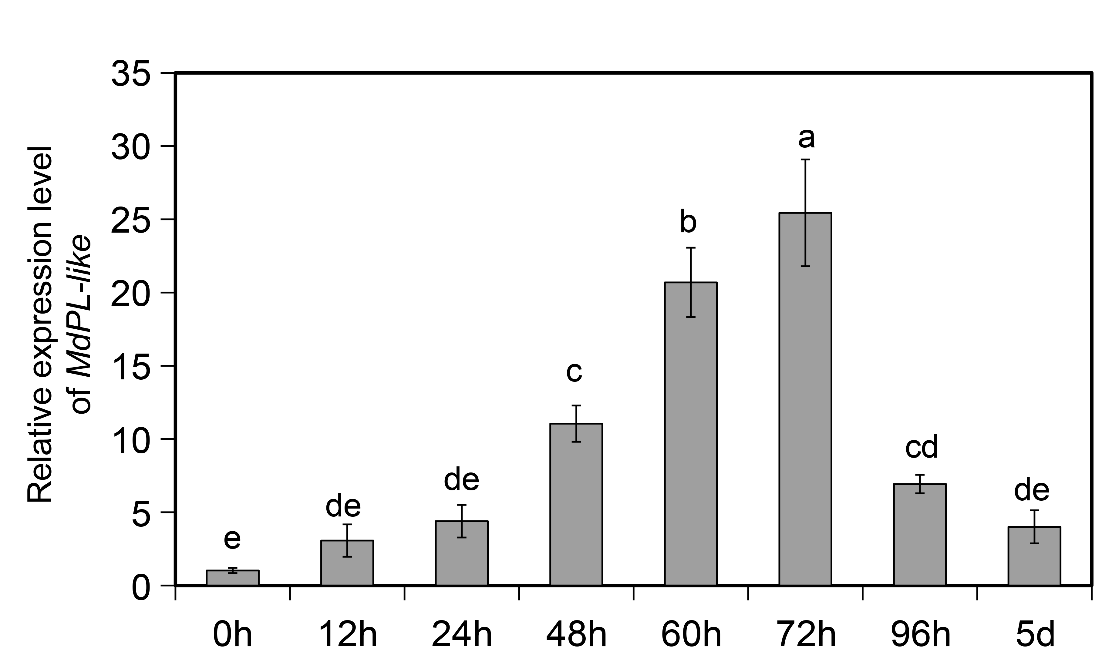

**Supplemental Figure 7**. **Relative expression level of *MdPL-like* after *C. fructicola* infection.** Error bars represent SD based on three biological replicates. Bars labeled with different letters indicate values that are significantly different at *P* <0.05 based on one-way ANOVA and Duncan’s test.

Table S1. The primers used in this study.

| Annotation | Name | Primer sequences |
| --- | --- | --- |
| Gene clone | PMD-MdWRKY17 | F:ATGACTTCTTCCTTCACTCACCT |
|  | MD12G1181000 | R:TCAGAACTCAGAAAATCCATAACT |
|  | PMD-MdVQ17 | F:ATGGAACCATTAAGATCTTATTC |
|  | MDP0000856686 | R:CTAATAGCTCGAGTCCACCGTGC |
| Subcellular | GFP-MdVQ17 | F:CGAGCTCGGTACCCGGGGATCCATGGAACCATTAA |
| localization |  | R:CCTTGCTCACCATGGTGTCGACATAGCTCGAGTCC |
| Y2H  analysis | pGAD424-MdVQ17 | F:AGATCGAATTCCCGGGGATCCGTATGGAACCATTA |
|  |  | R:CATAGATCTCTGCAGGTCGACCTAATAGCTCGAGT |
|  | pGAD424-MdVQ17 | F:AGATCGAATTCCCGGGGATCCGTTCGTTGAAGCCA |
|  | △1-50 | R:CATAGATCTCTGCAGGTCGACCTAATAGCTCGAGT |
|  | pGAD424-MdVQ17 | F:AGATCGAATTCCCGGGGATCCGTTCGTTGAAGCCA |
|  | △1-50△111-161 | R:CATAGATCTCTGCAGGTCGACGATATCGATCAGTG |
|  | pGAD424-MdVQ17 | F:AGATCGAATTCCCGGGGATCCGTTCGTTGAAGCCA |
|  | △1-50△90-161 | R:CATAGATCTCTGCAGGTCGACGCAGGTGAGGCGCTG |
|  | pGAD424-MdVQ17 | F:AGATCGAATTCCCGGGGATCCGTTTCAGGGAGCTTG |
|  | △1-81△111-161 | R:CATAGATCTCTGCAGGTCGACGATATCGATCAGTGG |
|  | pGBT9-MdWRKY17 | F:CGCCGGAATTCCCGGGGATCCGTAATGGTGCTCCA |
|  | △1-190 | R:TTAGCTTGGCTGCAGGTCGACTCAGAACTCAGAAAA |
|  | pGBT9-MdWRKY17 | F:CGCCGGAATTCCCGGGGATCCGTAATGGTGCTCCA |
|  | △1-190△470-512 | R:TTAGCTTGGCTGCAGGTCGACTCATGGCAAGGGCCT |
|  | pGBT9-MdWRKY17 | F:CGCCGGAATTCCCGGGGATCCGTCAGTCTACAAGA |
|  | △1-272△470-512 | R:TTAGCTTGGCTGCAGGTCGACTCATGGCAAGGGCCT |
|  | pGBT9-MdWRKY17 | F:CGCCGGAATTCCCGGGGATCCGTAATGGTGCTCCA |
|  | △1-190△384-512 | R:TTAGCTTGGCTGCAGGTCGACTCACCTATACCCGTC |
| Protein  purification | pET-32a-MdWRKY17 | F:GCCATGGCTGATATCGGATCCATGACTTCTTCCT |
|  |  | R:CTCGAGTGCGGCCGCAAGCTTGGAACTCAGAAAAT |
|  | pET-32a-MdVQ17 | F:GCCATGGCTGATATCGGATCCATGGAACCATTAA |
|  |  | R:CTCGAGTGCGGCCGCAAGCTTGCTAATAGCTCGAG |
|  | pMAL-c5x-MdVQ17 | F:GAGGGAAGGATTTCACATATGATGGAACCATTAA |
|  |  | R:ACCTGCAGGGAATTCGGATCCCTAATAGCTCGAG |
| BIFC  analysis | pSPYNE-MdWRKY17 | F:TGGCGCGCCACTAGTGGATCCATGACTTCTTCCTTATGACTTCTTCCT |
|  |  | R:AGCGGTACCCTCGAGGTCGACGAACTCAGAAAATGAACTCAGAAAAT |
|  | pSPYCE-MdVQ17 | F:TGGCGCGCCACTAGTGGATCCATGGAACCATTAAGATGGAACCATTA |
|  |  | R:AGCGGTACCCTCGAGGTCGACATAGCTCGAGTCCAATAGCTCGAGTCCA |
| Split-LUC  analysis | nLUC-MdWRKY17 | F:GGGGACAAGTTTGTACAAAAAAGCAGGCTTCATGACTTCTTCCT |
|  |  | R:GGGGACCACTTTGTACAAGAAAGCTGGGTTGAACTCAGAAAAT |
|  | cLUC-MdVQ17 | F:GGGGACAAGTTTGTACAAAAAAGCAGGCTTCATGGAACCATTAA |
|  |  | R:GGGGACCACTTTGTACAAGAAAGCTGGGTTCTAATAGCTCGA |
|  | cLUC-MdWRKY17 | F:GGGGACAAGTTTGTACAAAAAAGCAGGCTTCATGACTTCTTCCT |
|  |  | R:GGGGACCACTTTGTACAAGAAAGCTGGGTTTCAGAACTCAGAA |
|  | nLUC-MdVQ17 | F:GGGGACAAGTTTGTACAAAAAAGCAGGCTTCATGGAACCATTAA |
|  |  | R:GGGGACCACTTTGTACAAGAAAGCTGGGTTTATAGCTCGAGTCC |
| Y1H  analysis | pAbAi-MdPL-Pro | F:CTTGAATTCGAGCTCGGTACC CCGATGGTGCAAA |
|  |  | R:AGCACATGCCTCGAGGTCGAC TTTACTAGCTGAC |
|  | pGADT7-MdWRKY17 | F:GCCATGGAGGCCAGTGAATTCATGACTTCTTCCT |
|  |  | R:CAGCTCGAGCTCGATGGATCCTCAGAACTCAGAAA |
| Dual-luciferase  analysis | pGreen0800-MdPL | F: GTCGACGGTATCGATAAGCTT CCGATGGTGCA |
|  |  | R:CGCTCTAGAACTAGTGGATCC TTTACTAGCTG |
|  | pGreen0800-MdDMR6 | F: GTCGACGGTATCGATAAGCTT ATCCTCCAGTA |
|  |  | R: CGCTCTAGAACTAGTGGATCC TCAGTGGTGTT |
|  | SK62-MdWRKY17 | F: CGCTCTAGAACTAGTGGATCCATGACTTCTTCCT |
|  |  | R: GATAAGCTTGATATCGAATTCTCAGAACTCAGAA |
|  | SK62-MdVQ17 | F: CGCTCTAGAACTAGTGGATCCATGGAACCATTAA |
|  |  | R: GATAAGCTTGATATCGAATTCCTAATAGCTCGAG |
| RT-qPCR | qRT-PCR-MdMDH | F:CGTGATTGGGTACTTGGAAC |
|  |  | R:TGGCAAGTGACTGGGAATGA |
|  | qRT-PCR-MdWRKY17 | F:GACACCCAGCAAGGAATTAAGG |
|  |  | R:CCCGTCTTCTCAGCACTAAAAT |
|  | qRT-PCR-MdDMR6 | F:CCAGAACATTCAATCCCCATC |
|  |  | R:TGCTGAACTCCAACGAAACCT |
|  | qRT-PCR-MdPL | F:GCAACTGCACTTTCACTAAAGA |
|  |  | R:GAAATTGTTGGCTTGACGTTTG |
|  | qRT-PCR-MdVQ1 | F: CACCACCACCACCAACACTCAC |
|  |  | R: GGAAGAGGAAGCGGAGGAGGAG |
|  | qRT-PCR-MdVQ5 | F: CAACTCCAAGGTCCACGTCCAAC |
|  |  | R: TGGTTGTTGTGACGGTTGTGGTAC |
|  | qRT-PCR-MdVQ6 | F: TGGTGGTGGTGGTGGTGATGG |
|  |  | R: GAAGGAATCGGAGTAAGCGAAGCG |
|  | qRT-PCR-MdVQ7 | F: CAGCCGCCGCTACAAGTACAG |
|  |  | R: TGGAACACATCGTTACCACTACCG |
|  | qRT-PCR-MdVQ10 | F: CCACCACGCTGCTCAACACTG |
|  |  | R: TGCTGCTGAGGCTGTAGAGGATG |
|  | qRT-PCR-MdVQ11 | F: GTGTCGTAGGTGTAGGAGGAGAGG |
|  |  | R: GGCGGCATGAAGCAGGAACTC |
|  | qRT-PCR-MdVQ12 | F: CCACCTCCAGCTCCTCTTCCTG |
|  |  | R: GCGGCATTGGTGTAGGTGATGG |
|  | qRT-PCR-MdVQ15 | F: TGGCAGCTTCAGATGGCAATGATG |
|  |  | R: AGTCCACCGCACCTTCACCTC |
|  | qRT-PCR-MdVQ16 | F: CTCGCCTCCGTACACAAGACAAG |
|  |  | R: CCTCCTCCTCCGCCTCCTTG |
|  | qRT-PCR-MdVQ17 | F:TCTTCATCCTCCTCCACCACCAAG |
|  |  | R:GTGCAACCGCAGGCTTCTTCC |
| RT-qPCR | qRT-PCR-MdVQ18 | F: CACCAAGTACGTGGAGACGGATG |
|  |  | R: TTGTAATCGGCTCCTGCACTTCG |
|  | qRT-PCR-MdVQ19 | F: AACAGCAACCACCACCTCAACG |
|  |  | R: TGAAGGAGGAGGTGTCAGCTTGG |
|  | qRT-PCR-MdVQ20 | F: AAACCGCTAAGCAAGCCTCT |
|  |  | R: GATTGTTGCTTTGTGGGCGT |
|  | qRT-PCR-MdVQ21 | F: AATATTCACGACGGCTTCCAAGGC |
|  |  | R: CCATCATGGCTGCGCCTAACG |
|  | qRT-PCR-MdVQ24 | F: TCTTCCTCTTCAGCCTCCTACACC |
|  |  | R: CGAACCGAGTGGAGTGATGACTG |
|  | qRT-PCR-MdVQ25 | F: ATGGAGGAGGCTCAGACGCTAAC |
|  |  | R: TCCAGTGAGTCTCTGAACCAGCTC |
|  | qRT-PCR-MdVQ27 | F: GTCCAAGGCCAGCACCTCTT |
|  |  | R: TCCCAGTAAGCCGTTGAACCA |
|  | qRT-PCR-MdVQ28 | F: GCCTGCACCTCTTATGGTGA |
|  |  | R: GTGAGCCGTTGAACCAAACC |
|  | qRT-PCR-MdVQ30 | F: CACCAAGTACGTGGAGACGGATG |
|  |  | R: TTGTAACTGGCTCCTGCACTTCG |
|  | qRT-PCR-MdVQ31 | F: TCAAGCTGCAAGAGCGAAGACAC |
|  |  | R: AGACGACGGCGACTCGGAAC |
|  | qRT-PCR-MdVQ33 | F: ACAAAAGGCGATGGACGTGC |
|  |  | R: TGAGCTCCTGGACAAGTGCC |
|  | qRT-PCR-MdVQ34 | F: TGAAGAGGAAGATCGAGCCATTGC |
|  |  | R: GCTCAGGAGTGTGGTCAGTCATTC |
|  | qRT-PCR-MdVQ35 | F: TGCTTAATGGCGTCGTCAGAGAAC |
|  |  | R: ATCCAGGAGTCGGCGAAGGTG |
|  | qRT-PCR-MdVQ36 | F: GAACGTGCTCGGTGTGAACG |
|  |  | R: AGCTCCTGAACAAGTGCCCT |
|  | qRT-PCR-MdVQ37 | F: GGTGGCGGAGGAGGACAAGG |
|  |  | R: AGGAGGACGGCAGAGACTTGTG |
|  | qRT-PCR-MdVQ41 | F: GGTGGCGGCTTCTTCTTCGAC |
|  |  | R: TTGGAATGGAAGGTGGTGGTTGTG |
|  | qRT-PCR-MdVQ42 | F: GTCCAAGCTGACTCCTCGAACTTC |
|  |  | R: GTGGTGGTGGTGGCTGTTGTG |
|  | qRT-PCR-MdVQ47 | F: AGCTCACCGGCCAAGACTCC |
|  |  | R: GGAAGCCACCTCTCGATCTAATGC |
| EMSA probes | MdDMR6 | F: ATATTGTTTTCTGCATTTTTGACTTAATTTTTATAAATTAA |
|  |  | R: TTAATTTATAAAAATTAAGTCAAAAATGCAGAAAACAATAT |
|  | MdDMR6-mut | F: ATATTGTTTTCTGCATTTTTAACTTAATTTTTATAAATTAA |
|  |  | R: TTAATTTATAAAAATTAAGTTAAAAATGCAGAAAACAATAT |
|  | MdPL-like-P1 | F: AGGAGTACCTTTGGTCAAACTATATTCAAA |
|  |  | R: TTTGAATATAGTTTGACCAAAGGTACTCCT |
| EMSA probes | MdPL-like-P2 | F: AAGTCAACTAAAAAAGTCAACGTCTGACCGTTGACTGGTCAAAG |
|  |  | R: CTTTGACCAGTCAACGGTCAGACGTTGACTTTTTTAGTTGACTT |
|  | MdPL-like-P3 | F: AGTTGACCATTGACCGTTGACTGAGTCAACAGTCAAGGTCAACT |
|  |  | R: AGTTGACCTTGACTGTTGACTCAGTCAACGGTCAATGGTCAACT |
|  | MdPL-like-P1-mut | F: AGGAGTACCTTTAAAAAAACTATATTCAAA |
|  |  | R: TTTGAATATAGTTTTTTTAAAGGTACTCCT |
|  | MdPL-like-P2-mut | F: AATTTAACTAAAAAATTTAACGTCTGACCGTTAAATGGTTAAAG |
|  |  | R: CTTTAACCATTTAACGGTCAGACGTTAAATTTTTTAGTTAAATT |
|  | MdPL-like-P3-mut | F: AGTTAAAAATTAATTGTTAATTGATTTAACATTTAATTTTAACT |
|  |  | R: AGTTAAAATTAAATGTTAAATCAATTAACAATTAATTTTTAACT |
| Chip-qPCR  analysis | MdDMR6-pro | qF: GTGATTTTCGTTTTCCTTCATTATTC |
|  |  | qR: ATACTGTGATATTCTTACACTCAGACCAT |
|  | MdPL-like-pro1 | qF: CGGAATCACCGTTAATAATT |
|  |  | qR: CCGTTTTTGAAGTCCGTTTT |
|  | MdPL-like-pro2 | qF: GTTCAGGACGTCGAAATTAG |
|  |  | qR: TTTTGACTTTGACCAGTCAA |
|  | MdPL-like-pro3 | qF: TCAACTTTGACAGTTGACCA |
|  |  | qR: TTCGGCCCAACCCAATCCAA |
| Genetic  transformation | pCambia2300-MdVQ17 | F: GAGAACACGGGGGACTCTAGAATGGAACCATTA |
|  |  | R: GGGAAATTCGAGCTCGGTACCCTAATAGCTCGA |
|  | pK7GWIWG2D-MdVQ17 | F: GGGGACAAGTTTGTACAAAAAAGCAGGCTTCCCTCTTCATCCTC |
|  |  | R: GGGGACCACTTTGTACAAGAAAGCTGGGT GATTCTTGGTGGGGT |
|  | pCambia2300GFP-MdPL | F: CGAGCTCGGTACCCGG GGATCC ATGGAGGGGACTT |
|  |  | R: CCTTGCTCACCATGGT GTCGACGATCAAGGGAGGA |
|  | pK7GWIWG2D-MdPL | F: GGGGACAAGTTTGTACAAAAAAGCAGGCTTCGAGGATATTATCA |
|  |  | R: GGGGACCACTTTGTACAAGAAAGCTGGGTTTGAAGATGACAT |
|  | pGWB415-MdWRKY17 | F: GGGGACAAGTTTGTACAAAAAAGCAGGCTTCATGACTTCTTCCT |
|  |  | R: GGGGACCACTTTGTACAAGAAAGCTGGGTTTCAGAACTCAGAA |
